# Lobophorin Producing Endophytic *Streptomyces olivaceus* JB1 Associated With *Maesa japonica* (Thunb.) Moritzi & Zoll.

**DOI:** 10.3389/fmicb.2022.881253

**Published:** 2022-04-29

**Authors:** Soohyun Um, Jaeyoun Lee, Seung Hyun Kim

**Affiliations:** College of Pharmacy, Yonsei Institute of Pharmaceutical Sciences, Yonsei University, Incheon, South Korea

**Keywords:** actinobacteria, *Streptomyces*, endophyte, symbiosis, lobophorin, antimicrobial

## Abstract

In this study, we focused on endophytes of *Maesa japonica* (Thunb.) Moritzi & Zoll. and the plant-microbe interaction at metabolite levels. We isolated seven endophytes associated with *M*. *japonica* (JB1−7), and focused on *Streptomyces olivaceus* JB1 because of antibacterial activities of its secondary metabolites. We confirmed lobophorin analogs production from the bacterial strain JB1 by using spectroscopic techniques such as NMR, UV, and LC/Q-TOF-MS. In the LC/MS system, thirteen reported lobophorin analogs and twelve unreported analogs were detected. Among metabolites, lobophorin A was clearly detected in the dried foliar residues of *M. japonica* which implies that JB1 resides in the host and accumulates its secondary metabolites likely interacting with the plant. Antimicrobial activity tests of the secondary metabolites against undesirable contaminants isolated from the external surface of *M*. *japonica* supported the host and microbe mutualistic relationship. In the meantime, lobophorin producing *Streptomyces* spp. were isolated from marine environments such as marine sediments, algae, corals, and sponges. As lobophorin producing *Streptomyces* is isolated commonly from marine environments, we conducted a saline water stress tolerance test with JB1 showing saline medium does not accelerate the growth of the bacterium.

## Introduction

*Maesa japonica* (Thunb.) Moritzi & Zoll. of the monotypic family Maesaceae is a perennial evergreen shrub growing up to 1–1.5 m that is geographically distributed throughout humid subtropical climate regions such as South-Central China, Southeast China, Japan, Taiwan, and Vietnam. In South Korea, the previously unrecorded *M*. *japonica* was first discovered in 2006 and thrives within a specific wet shrub forest area across southwestern Jeju (Supplementary Figure 12). *M*. *japonica* has been used as a medicinal plant in certain parts of China and has high potential pharmaceutical value because of its rich secondary metabolites, including maesaquinone, embelin, rapanone, and 1,1-diphenyl-2-picrylhydrazyl (DPPH), which act as antioxidants (Moon et al., 2006; Kim, 2014; Kwon et al., 2020). Recently, *M*. *japonica* has also been recognized as an indoor air purifying plant that removes particulate matter (PM) and reduces fine dust including PM1, PM2.5, and PM10 (Kwon et al., 2021). Despite these unique advantages of *M*. *japonica*, its phyllosphere endophytes have remained unstudied due to its low accessibility and non-agricultural uses.

In comparison to deciduous plants whose leaves basically have an annual lifespan, the longer leaf lifespan of perennial evergreen shrubs could allow the establishment of a relatively more stable year-round microbial community within the phyllosphere. For example, a broad-leaf evergreen plant such as southern magnolia, *Magnolia grandiflora*, combining a typical leaf surface with the presence of year-round leaves proves to be an effective plant-microbe ecosystem, little influenced by environmental change and seasonal variation in its phyllosphere communities and displaying no major changes in bacterial lineages (Jackson and Denney, 2011). Additionally, Müller et al. (2015) reported that the endophytic bacterial diversity of olive trees, a representative evergreen species, may be stable over long periods of time because of the longevity and high genetic variability of cultivars; and these organisms may have antagonistic potential against the fungus *Verticillium dahliae* (Aranda-Ocampo et al., 2011). Human et al. (2016) detected antifungal secondary metabolites such as antifungal fungichromin, and antiviral actiphenol from endophytic *Streptomyces* spp. of *Protea* infructescences, an evergreen perennial plant distributed throughout sub-Saharan Africa and identified *Streptomyces* spp. as the most likely organism contributing diverse advantages to *Protea* infructescences by maintaining the plant-microbe symbiosis.

This study reports bacterial and fungal endophytes from *M*. *japonica* and antibiotic metabolites produced by *Streptomyces olivaceus* as endophytes in nature. The *S. olivaceus* sp. is known as a marine-derived bacterial strain isolated from diverse marine environments. The strain of *S*. *olivaceus* SCSIO T05 was isolated from an Indian Ocean deep-sea sediment producing rishirilides, galvaquinones, and lupinacidin D (Zhang et al., 2018) and the *S*. *olivaceus* OUCLQ19-3 was isolated from a mud sample collected from the cold-seep area of the South China Sea producing antibacterial dixiamycins (Jin et al., 2021). The *S*. *olivaceus* FXJ8.012 producing tetroazolemycins and spoxazomicins was isolated from an Indian Ocean deep-sea water sample (Liu et al., 2013). In recent years, the *S*. *olivaceus* sp. as producers of lobophorins were isolated from marine sediment (Chen et al., 2013; Nguyen et al., 2020) and the *S*. *olivaceus* sp. associated with the cold-water coral *Lophelia pertusa* produces our target metabolites, lobophorin analogs (Braña et al., 2017).

We focused on a specific hypothesis that unrevealed beneficial endophytes of the host, *M*. *japonica*, a perennial evergreen shrub, produce antibiotic metabolites that are accumulated *in planta* at detectable levels. The antimicrobial activities of secondary metabolites of the *S*. *olivaceus* JB1 (GenBank accession no. OM845781, 99%) against external strains of known phytopathogen strains, including four bacterial strains, *Bacillus megaterium*, *Bacillus thuringiensis*, *Leclercia adecarboxylata*, and *Pseudomonas punonensis*, and one fungal strain, *Mucor circinelloides* were also demonstrated.

## Materials and Methods

### Plant Collection, Bacterial Isolation, and Cultivation Procedures

A vegetative shoot of the *M. japonica* was initially collected as a plant material from an unmanned forest (Hwasun Gotjawal, N33.2636198 E126.3346747) in Jeju Island, South Korea in April 2021. The foliage of *M*. *japonica* was soaked in 5% sodium hypochlorite for 5 min and the surface was disinfected with 80% aqueous ethanol to remove external contaminants from the leaf blade. The sterilized leaves and leaf residues of *M*. *japonica* were cut into pieces and placed onto chitin medium (6 g of chitin, 0.75 g of K_2_HPO_4_, 0.5 g of MgSO_4_⋅7H_2_O, and 3.5 g of K_2_HPO_4_, 10 mg of FeSO_4_⋅7H_2_O, 10 mg of MnCl_2_⋅4H_2_O, 10 mg of ZnSO_4_⋅7H_2_O, 100 mg of cycloheximide, and 36 g of agar per 1 L of sterilized water), Czapek Dox medium (30 g of sucrose, 2 g of NaNO_3_, 1 g of K_2_HPO_4_, 0.5 g of MgCl, 0.5 g of KCl, 0.01 g of FeCl_2_, 100 mg of cycloheximide, and 18 g of agar per 1 L of sterilized water), and A1 medium (10 g of starch, 4 g of yeast extract, 2 g of peptone, 100 mg of cycloheximide, and 18 g of agar per 1 L of sterilized water) for 20 days to isolate endophytes associated with *M*. *japonica*. Identical bacterial isolation media without cycloheximide were used for isolating external microbes from *M*. *japonica*. Each isolate was cultivated in modified K media (12 g of LB, 12 g of PDB, 1 g of TSB, and 18 g of agar per 1 L of sterilized water, 28°C, 6 days).

DNA was extracted from the isolates by using a QIAamp DNA Mini Kit (Qiagen) according to the manufacturer’s instructions. The DNA extract was amplified with 10 pmole/μL primers, 2.5 mM dNTP mixture, 10x Taq PCR buffer 2 μL, template 20 ng/μL, KOMA-Taq 2.5 U/μL and distilled water. The amplification conditions were as follows: initial denaturation at 95°C for 5 min, followed by 30 cycles of denaturation at 95°C for 30 s, annealing at 55°C, and extension at 68°C for 1.5 min, and a final extension at 68°C for 10 min. The purified PCR products were sequenced in the forward and reverse directions in separate reactions and in duplicate. The PCR amplification primer sequences were as follows: 27F 5′ (AGA GTT TGA TCM TGG CTC AG) 3′ and 1492R 5′ (TAC GGY TAC CTT GTT ACG ACT T) 3′ for 16S rRNA. The primer sequences were as follows: NS1 5′ (GTA GTC ATA TGC TTG TCT C) 3′ and NS24 5′ (AAA CCT TGT TAC GAC TTT TA) 3′ for 18S rRNA. The purified PCR products were sequenced by using two primer pairs, 785F 5′ (GGA TTA GAT ACC CTG GTA) 3′ and 907R 5′ (CCG TCA ATT CMT TTR AGT TT) 3′ for 16S rRNA and NS1 5′ (GTA GTC ATA TGC TTG TCT C) 3′ and NS24 5′ (AAA CCT TGT TAC GAC TTT TA) 3′ for 18S rRNA. Sequencing was performed by using a Big Dye terminator cycle sequencing kit v.3.1 (Applied Biosystems, United States). Sequencing products were resolved on an Applied Biosystems model 3730XL automated DNA sequencing system (Applied Biosystems, United States).

### Bacterial Metabolomic Studies

After cultivating the isolates JB1−JB7 in modified K liquid media for 6 days, the cultures were subjected to extraction with ethyl acetate/water layer separation by a separating funnel, and the organic phase was concentrated *in vacuo*. The crude extract was dissolved in methanol at a concentration of 250 μg/mL and analyzed with HPLC (high-performance liquid chromatography) − MS. HPLC measurements were carried out on an Agilent 1290 UHPLC system (Agilent Technologies, Santa Clara, CA, United States) equipped with a 1290 Infinity binary pump and YMC-triart C18 column (150 × 2.0 mm, 1.9 μm; YMC Korea Co., Seongnam, South Korea). Each sample was analyzed by using the following conditions: gradient: 0-20 min, 10-100% B, 20-23 min, 100% B, 23-24 min, 100-10% B, 24-30 min, 10% B, with an injection volume of 10 μL and a flow rate of 0.4 mL/min. All eluents of the HPLC system were acidified with 0.1% formic acid. H_2_O was used as eluent A, while acetonitrile served as eluent B. Mass spectrometry was performed on an Agilent 6530 quadrupole-time of flight mass spectrometer (Q-TOF-MS, Agilent Technologies, Santa Clara, CA, United States) equipped with an electrospray ion (ESI) source. The MS experiment was performed under the following conditions: drying gas temperature 300°C, drying gas flow rate 8 L/min, sheath gas temperature 350°C, sheath gas flow rate 11 L/min, and capillary voltage +3.5 kV with positive mode (pressure of nebulizer 35 psig, capillary voltage 3500 V, fragmentor 175 V, skimmer 65 V, and OCT 1 RF Vpp 750 V).

### Impact of Saline Water Stress Tolerance on *S. olivaceus* JB1

A 10 mL preculture of the bacterial strain JB1 was transferred into non-saline and saline K media (salinity; 33 per mille of sodium chloride) separately. Both cultures were cultivated under the same conditions at 28°C with shaking at 180 rpm for 4 days. Bacterial cell growth rates were compared based on the measurement of optical density (OD) and weighing of the centrifuged bacterial cells (4000 rpm, 10 min, Centrifuge 5810 R, Eppendorf, Hamburg, Germany) after removing the supernatant of the bacterial cultures of *S*. *olivaceus*. Additionally, a metabolomic comparison of *S*. *olivaceus* JB1 from saline and non-saline media was performed after analyzing secondary metabolites of the bacterial strain JB1 with LC/Q-TOF-MS. The analyses were performed with a 1290 Infinity binary pump and YMC-triart C18 column (150 × 2.0 mm, 1.9 μm; YMC Korea Co., Seongnam, South Korea). The LC/MS conditions were as follows: 0-20 min, 10-100% B, 20-23 min, 100% B, 23-24 min, 100-10% B, 24-30 min, 10% B, 30-35 min with an injection volume of 20 μL and a flow rate of 0.4 mL/min. Eluent A of the HPLC system was acetonitrile acidified with 0.1% formic acid, and eluent B was H_2_O acidified with 0.1% formic acid. Mass spectrometry was performed on an Agilent 6530 Q-TOF-MS (Agilent Technologies, Santa Clara, CA, United States) with an electrospray ion (ESI) source.

### Analyses of Secondary Metabolites From *S. olivaceus* JB1

The modified K Medium was used for HRMS^2^-based GNPS (Global Natural Product Social Molecular Networking) analysis of ethyl acetate crude extracts obtained from 1-week-old cultures of *S*. *olivaceus* JB1. Based on the analyzed tandem mass (MS^2^) data from an Agilent 6530 Q-TOF-MS, the molecular networks of the secondary metabolites were created by using the GNPS platform^1^. The data was converted to the .*mzML* format with MS-Convert. The converted files were used to generate an MS/MS molecular network using the GNPS web-server. The precursor ion mass tolerance was set to 2.0 Da and the product ion tolerance was set to 0.05 Da. The molecular networks were generated using a minimum of 6 matched peaks, a cosine score of 0.7 and a minimum cluster size of 2-3. After analysis, data were visualized using Cytoscape 3.8.2 software (Shannon et al., 2003). A literature investigation on chemical structures of lobophorin analogs and their biological function was conducted based on SciFinder-n and AntiBase comparing with the acquired combinational data of MS, UV, and elution times on LC/MS of individual lobophorin analogs.

### Identification of Lobophorins A and G From *S. olivaceus* JB1

A 10 mL aliquot of *Streptomyces olivaceus* JB1 preculture was transferred to a 250 mL Erlenmeyer flask containing 100 mL of modified K medium. With continuous shaking (28°C, 180 rpm, 7 days), 10 mL of the liquid culture was inoculated in a 2 L Erlenmeyer flask containing 1.2 L of the medium (28°C, 180 rpm, 6 days, 48 L in total). Then, the culture was extracted by using ethyl acetate/water layer separation, and the ethyl acetate layer was concentrated *in vacuo*. The dried extract was loaded onto a prepacked SPE-C18 column (S*Pure, Singapore) and stepwise-eluted (20, 40, 60, 80, and 100% aqueous methanol). The lobophorin analogs were eluted with 80% aqueous methanol, and the fraction was further purified with semipreparative HPLC equipped with a 1260 Infinity binary pump and YMC-triart C18 column (150 × 10.0 mm, 5 μm; YMC Korea Co., Seongnam, South Korea) using the following conditions: gradient: 0–60 min, 10–90% B, 61–80 min, 90% B with an injection volume of 200 μL and a flow rate of 3 mL/min, UV detection at 204 and 268 nm. All eluents of the HPLC system were acidified with 0.1% formic acid. H_2_O was used as eluent A, while acetonitrile served as eluent B. NMR data of the separated single compounds, lobophorin A and G, were recorded on Bruker 700 and 800 MHz NMR spectrometers equipped with a Bruker CryoPlatform at the Korea Basic Science Institute in Ochang, South Korea. ^1^H NMR chemical shifts at δ_*H*_ 7.24, 3.31, and 2.50 (CDCl_3_, CD_3_OD, in a ratio of 8:3 and DMSO-*d*_6_) and ^13^C NMR chemical shifts at δ_*C*_ 77.2, 49.1, and 49.0 (CDCl_3_, CD_3_OD in a ratio of 8:3 and DMSO-*d*_6_) were set as reference peaks, and the chemical structures of lobophorins A and G were assigned by interpreting combinational data from correlation spectroscopy (COSY), heteronuclear single quantum coherence (HSQC), and heteronuclear multiple bond correlation (HMBC) experiments.

### Lobophorin Detection in a Botanical Specimen

The collected fresh foliage of *M. japonica* was dried at 50°C for 24 h with a dehydrator (Shinil, Cheonan, South Korea). The dried leaves and leaf residues (6.0 g in total) were ground into a powder, and then the powder was extracted with 60 mL methanol at 40°C for 60 min with an ultrasound-assisted extraction method. The methanol extract was concentrated *in vacuo* and dissolved in methanol at a concentration of 250 μg/mL. Metabolite analysis was conducted with MS/Q-TOF-MS with the following conditions: gradient: 0–20 min, 10–100% B, 20–23 min, 100% B, 23–24 min, 100–10% B, 24–30 min, 10% B, with an injection volume of 10 μL and a flow rate of 0.4 mL/min. All eluents of the HPLC system were acidified with 0.1% formic acid. H_2_O was used as eluent A, while acetonitrile served as eluent B. Mass spectrometry was performed with an electrospray ion (ESI) source in positive mode. Extracted ion current (EIC) chromatograms (*m*/*z* [M + H]^+^ and *m*/*z* [M + Na]^+^) and fragmentation patterns were analyzed to compare and detect lobophorin analogs in both the methanol extract of *M*. *japonica* and the liquid culture broth of *S*. *olivaceus* JB1.

### Paper Disk Diffusion Assay

To isolate the external contaminants of *M. japonica*, the fresh foliage was placed onto K solid medium in the absence of cycloheximide for 20 days, and then four gram-negative bacterial and one fungus strains were isolated. For the paper disk diffusion assay on agar plates against contaminants, K agar medium was prepared in 90-mm-diameter Petri dishes. A total of 500 μL of each bacterial culture [*Bacillus megaterium* (JB8), *B. thuringiensis* (JB9), *L. adecarboxylata* (JB10), and *P. punonensis* (JB11)] was inoculated onto a K agar plate, evenly spread with cell spreaders and then incubated at 28°C for 3 days. The colonies of the fungus strain [*M. circinelloides* (JB12)] were inoculated in the center of the K agar medium plate and incubated at 28°C for 7 days. Three 6-mm-diameter sterile paper disks were placed onto the surface of each Petri dish and then imbued with the fractions of cultures of the gram-positive bacterial strain *S. olivaceus* (JB1) (1 mg/mL, 20 μL). Ciprofloxacin (1 mg/mL, 20 μL) and amphotericin B (1 mg/mL, 20 μL) were used as positive controls for the antibacterial and antifungal assays, respectively. A total of 20 μL of DMSO was added to every Petri dish as a negative control.

## Results

### Isolation of Microbes Associated With *M. japonica* and DNA Sequencing

**FIGURE 1:**
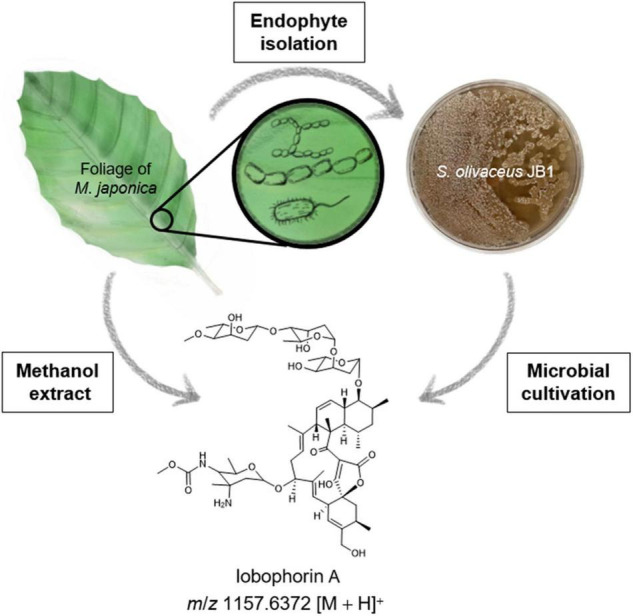
Schematic overview of isolation of endophytes associated with *M*. *japonica* and lobophorin detection from the plant material and the endophytic bacterium *S*. *olivaceus* JB1. The bacterial strain *S. olivaceus* JB1 was isolated from the sterilized lateral vein of *M. japonica, S. olivaceus* JB1 was cultured in modified K liquid medium for 6 days, and liquid culture broth was extracted by using ethyl acetate/water layer separation. The organic phase was concentrated *in vacuo*. The foliage and petioles of *M. japonica* were subjected to extraction with methanol. The extracts were analyzed using Q-TOF-MS, and notably, lobophorin A (*m*/*z* 1157.6372 [M + H]^+^) was detected from both the *S. olivaceus* JB1 extract and *M. japonica.*

**FIGURE 2:**
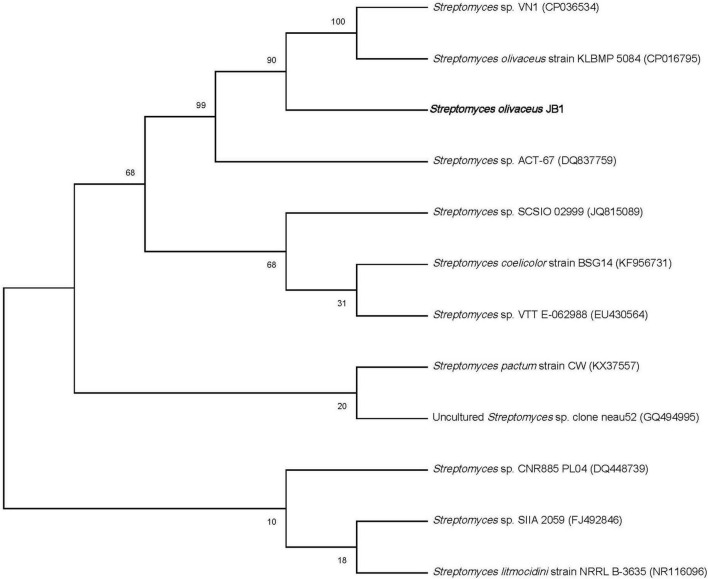
Maximum-likelihood phylogenetic tree showing the phylogenetic relationship of *S. olivaceus* JB1 and other closely related species based on 16S rRNA gene sequences.

From the well-disinfected foliar residues of *M. japonica*, a total of seven putative endophytes were obtained, including one gram-positive bacterium (JB1), four gram-negative bacteria including *Enterobacter ludwigii* (JB2) (GenBank accession no. OM845779, 99%), *Pseudomonas koreensis* (JB3) (GenBank accession no. OM845778, 99%), *Pseudomonas aeruginosa* (JB4) (GenBank accession no. OM845780, 99%), and *Enterobacter cloacae* (JB5) (GenBank accession no. OM838295, 99%), and two fungi [JB6 (GenBank accession no. OM845784, 99%) and JB7 (GenBank accession no. OM845783, 99%)]. Only the bacterial strain JB1 showed a typical *Streptomyces* morphological feature, namely, the formation of mycelium covered with white spores (Figure 1). The bacterial strain JB2-5 displayed typical gram-negative bacterial characteristics. The aligned 16 rRNA dataset and phylogenetic analyses identified one Actinobacterium *Streptomyces* sp. (Figure 2). Four gram-negative bacteria were identified as common bacterial endophytes, including *Enterobacter ludwigii* (JB2) known as tomato growth-promoting endophyte (Bendaha and Belaouni, 2020), and *Pseudomonas koreensis* (JB3) studied as tobacco plants endophyte reducing bacterial wilt disease (Shang et al., 2021). Also, *Pseudomonas aeruginosa* (JB4) and *Enterobacter cloacae* (JB5) were known as *Achyranthes aspera* growth-stimulating endophyte (Devi et al., 2017), and banana plant growth supporting endophyte (Macedo-Raygoza et al., 2019), respectively. Two fungi, JB6-7 (identical species), were identified *Colletotrichum musae*, endophytic fungi from wild banana (Nuangmek et al., 2001; Bendaha and Belaouni, 2020; Shang et al., 2021).

Additionally, the external microbes from the surface of *M*. *japonica* were isolated without the addition of antibiotics. The isolates were four gram-negative bacterial strains, *Bacillus megaterium* (JB8) (GenBank accession no. OM838304, 99%), *Bacillus thuringiensis* (JB9) (GenBank accession no. OM838312, 99%), *Leclercia adecarboxylata* (JB10) (GenBank accession no. OM838305, 99%), and *Pseudomonas punonensis* (JB11) (GenBank accession no. OM838294, 99%), and one fungus strain *Mucor circinelloides* (JB12) (GenBank accession no. OM845785, 99%).

### Metabolomics Study of *S. olivaceus* JB1

**FIGURE 3:**
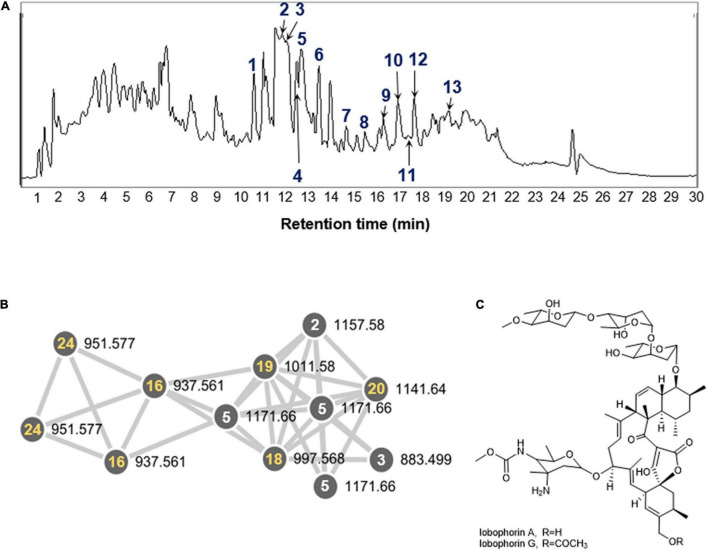
**(A)** LC-MS chemical analysis of lobophorin analogs in the extract of *S. olivaceus* JB1 and 25 annotated compounds. Thirteen compounds (**1**–**13**, *blue*) indicate previously reported lobophorin analogs. **(B)** A GNPS cluster assigned to lobophorin analogs from the organic phase of the *S. olivaceus* JB1 liquid culture. Five specific compounds (**16**, **18**, **19**, **20**, and **24**, *yellow*) correspond to unreported lobophorin analogs that clustered with reported lobophorin analogs in GNPS (selected). **(C)** The chemical structures of lobophorin A and G (**2** and **6**).

After cultivating the endophytes (JB1–7) associated with *M*. *japonica* in the modified K liquid and solid media to maximize the diversity of metabolites, the organic phase of the ethyl acetate/water layer separation was analyzed with the LC/MS system. The metabolite production from the isolates did not demonstrate prominent differentiation between either solid or liquid cultures. The bacterial strain JB1 showed broad mass peaks between *m*/*z* 883.5−1219.6 (Figure 3A and Supplementary Figure 13), displaying identical UV spectra (λ_*max*_ at 204, 268). A further literature study for the metabolites was performed with SciFinder (Schwall and Zielenbach, 2000) and AntiBase (Laatsch, 2017) and revealed that the major metabolites were lobophorin analogs belonging to a group of spirotetronate antibiotics. In accordance with the LC/MS chemical profiling, we annotated thirteen lobophorin analogs (**1**−**13**, Table 1 and Supplementary Figure 13). As we initially intended to investigate the antimicrobial secondary metabolites produced by the endophytes of *M*. *japonica* against external strains of known phytopathogen strains to verify this defensive symbiotic system, the predominant metabolites from *S*. *olivaceus* JB1, lobophorin A and G (Figure 3C, Table 1 and Supplementary Figure 13), were representatively isolated as single compounds with HPLC, and the chemical structures of **2** and **6** were confirmed based on NMR analysis (Supplementary Figures 2−11). HRMS^2^-based analysis of the ethyl acetate extract of *S*. *olivaceus* JB1 clustered structurally related molecules with GNPS, and the results were visualized by Cytoscape 3.9.0 (Figure 3B). Eight lobophorin analogs were clustered (**2**, **3**, **5**, **16**, **18**, **19**, **20**, and **24**), and the cluster represents these analogs have similar MS^2^ fragments. Among them, three nodes were reported for the lobophorin analogs lobophorin L (**2**), M (**3**), and E (**5**). Five nodes were unreported for the lobophorin analogs **16** (*m*/*z* 937.6 [M + H]^+^), **18** (*m*/*z* 997.6 [M + H]^+^), **19** (*m*/*z* 1011.6 [M + H]^+^), **20** (*m*/*z* 1141.7 [M + H]^+^), and **24** (*m*/*z* 951.6 [M + H]^+^).

**TABLE 1:** List of previously isolated lobophorins that detected from *S. olivaceus* JB1 and unknown compounds of *S. olivaceus* JB1.

RT^a^ (min)	*m*/*z*	Adduct	Compounds	Origin	Bacterial species	Bioactivities
10.7	1013.6	H^+^	Lobophorin L **(1)**	Marine sediment	*Streptomyces* sp. 4506	Antibacterial
11.9	1157.6	H^+^	Lobophorin A **(2)**	Algae-associated	*Streptomyces* sp. CNB-837	Antibacterial
12.0	883.5	H^+^	Lobophorin M **(3)**	Marine sediment	*Streptomyces* sp. 4506	Antibacterial
12.1	1185.6	H^+^	Lobophorin H **(4)**	Marine sediment	*Streptomyces* sp. 12A35	Antibacterial
12.2	1171.6	H^+^	Lobophorin E **(5)**	Marine sediment	*Streptomyces* sp. SCSIO 01127	Antibacterial
13.2	1199.6	H^+^	Lobophorin G **(6)**	Marine sediment	*Streptomyces* sp. MS100061	Antibacterial
14.4	1174.6	H^+^	Lobophorin K **(7)**	Coral-associated	*Streptomyces* sp. M-207	Antibacterial, cytotoxicity
15.3	1180.6	Na^+^	Lobophorin CR1 **(8)**	Marine sediment	*Streptomyces* sp. 7790_N4	Antibacterial, cytotoxicity
16.6	1209.6	Na^+^	Lobophorin C **(9)**	Sponge-associated	*Streptomyces carnosus* AZS17	Antibacterial, cytotoxicity
16.7	913.4	H^+^	Lobophorin I **(10)**	Marine sediment	*Streptomyces* sp. 12A35	Antibacterial
17.2	1219.6	H^+^	Lobophorin CR3 **(11)**	Marine sediment	*Streptomyces* sp. 7790_N4	Cytotoxicity
17.9	1203.6	H^+^	Lobophorin CR2 **(12)**	Marine sediment	*Streptomyces* sp. 7790_N4	Cytotoxicity
19.3	1049.5	Na^+^	Lobophorin F **(13)**	Marine sediment	*Streptomyces* sp. SCSIO 01127	Antibacterial, cytotoxicity
10.3	1205.7	H^+^	Unknown **(14)**	Terrestrial plants associated	*Streptomyces olivaceus* JB1	
10.8	1143.6	H^+^	Unknown **(15)**	Terrestrial plants associated	*Streptomyces olivaceus* JB1	
10.9	937.6	H^+^	Unknown **(16)**	Terrestrial plants associated	*Streptomyces olivaceus* JB1	
12.3	897.5	H^+^	Unknown **(17)**	Terrestrial plants associated	*Streptomyces olivaceus* JB1	
12.5	997.6	H^+^	Unknown **(18)**	Terrestrial plants associated	*Streptomyces olivaceus* JB1	
12.9	1011.6	H^+^	Unknown **(19)**	Terrestrial plants associated	*Streptomyces olivaceus* JB1	
13.7	1141.7	H^+^	Unknown **(20)**	Terrestrial plants associated	*Streptomyces olivaceus* JB1	
13.7	867.5	H^+^	Unknown **(21)**	Terrestrial plants associated	*Streptomyces olivaceus* JB1	
13.9	1257.6	H^+^	Unknown **(22)**	Terrestrial plants associated	*Streptomyces olivaceus* JB1	
14.2	1155.7	H^+^	Unknown **(23)**	Terrestrial plants associated	*Streptomyces olivaceus* JB1	
16.1	951.6	H^+^	Unknown **(24)**	Terrestrial plants associated	*Streptomyces olivaceus* JB1	
16.9	1193.6	Na^+^	Unknown **(25)**	Terrestrial plants associated	*Streptomyces olivaceus* JB1	

*^a^RT, retention time.*

### Impact of Saline Water Stress Tolerance on *S. olivaceus* JB1

**FIGURE 4:**
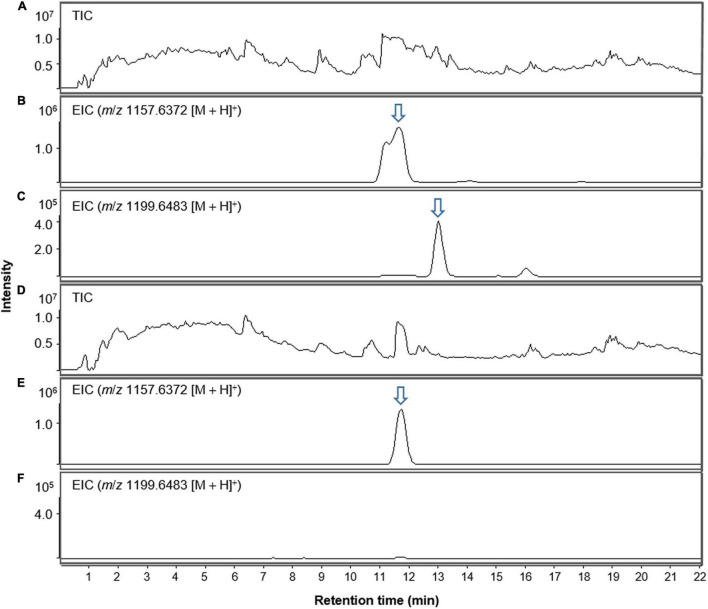
LC-MS chromatogram comparison of *S. olivaceus* JB1 culture extract in non-saline and saline media (salinity; 33 per mille of sodium chloride) in positive ion mode. **(A)** Total ion current (TIC) chromatogram of non-saline liquid culture broth of *S. olivaceus* JB1. **(B)** Extracted-ion chromatogram (EIC) of the non-saline liquid broth of *S. olivaceus* JB1 (lobophorin A, *m*/*z* 1157.6372 [M + H]^+^). **(C)** Extracted-ion chromatogram (EIC) of the non-saline liquid broth of *S. olivaceus* JB1 (lobophorin G, *m*/*z* 1199.6438 [M + H]^+^). **(D)** Total ion current (TIC) chromatogram of saline liquid culture broth of *S. olivaceus* JB1. **(E)** Extracted-ion chromatogram (EIC) of the saline liquid broth of *S. olivaceus* JB1 (lobophorin A, *m*/*z* 1157.6372 [M + H]^+^). **(F)** Extracted-ion chromatogram (EIC) of the saline liquid broth of *S. olivaceus* JB1 (lobophorin G, *m*/*z* 1199.6438 [M + H]^+^, *not detected*). The arrows indicate exact elution times of the extracted ions.

Interestingly, lobophorin-producing *Streptomyces* spp. have been isolated from marine environments such as marine sediment, algae−associated, coral−associated, and sponge−associated environments, whereas *S*. *olivaceus* JB1 was obtained from a terrestrial environment associated with *M*. *japonica* (Table 1). The growth and metabolite production of *S*. *olivaceus* JB1 were investigated by cultivating the strain in saline bacterial growth medium with 33 per mille of sodium chloride as a stressor to reveal whether *S*. *olivaceus* JB1 originated from a marine environment. JB1 was cultivated in saline media and non-saline media separately. Except for the differences in the salinity of the growth media, the cultivation conditions, such as the inoculum concentration and incubation temperature, were identical. JB1 grew more in the non-saline medium than in the saline medium (cell wet biomass in a ratio of 2.7:1.6 from 50 mL of the liquid cultures, 4 days, 180 rpm, centrifuged at 4,000 rpm, 10 min). Additionally, a comparison of the lobophorin A production rate in non-saline and saline media revealed that *S*. *olivaceus* JB1 produces 3.5 times more lobophorin A in non-saline medium. Unlike other lobophorin-producing *Streptomyces* spp., *S*. *olivaceus* JB1 showed differentiation in saline media, not accelerating its growth and secondary metabolite production (Figure 4 and Supplementary Figure 1).

*Streptomyces olivaceus* resides in certain plants as an endophytic bacterium in territorial environments. However, production of lobophorin analogs by endophytic *S*. *olivaceus* in a territorial environment has not been previously reported. Thus, the bacterial strain JB1 is the first example of a bacterium producing lobophorin analogs in a non-saline habitat.

### Lobophorin Detection in a Botanical Specimen

**FIGURE 5:**
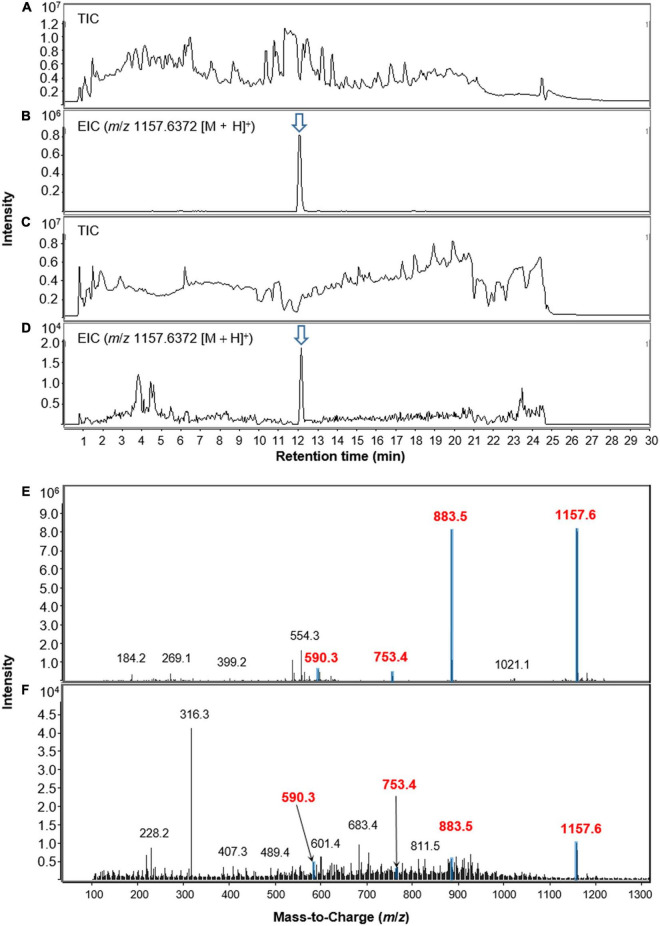
LC-MS chromatograms of lobophorin analogs obtained from extracts of *S. olivaceus* JB1 in broth medium and the foliage and petioles of *M*. *japonica* in positive ion mode. **(A)** Total ion current (TIC) chromatogram of liquid culture broth of *S. olivaceus* JB1. **(B)** Extracted-ion chromatogram (EIC) of the liquid broth of *S. olivaceus* JB1 (lobophorin A, *m*/*z* 1157.6372 [M + H]^+^). **(C)** Total ion current (TIC) chromatogram of the methanol extract of the dried leaf material of *M. japonica*. **(D)** Extracted-ion chromatogram (EIC) of methanol extract of *M. japonica* leaves (lobophorin A, *m*/*z* 1157.6372 [M + H]^+^). **(E)** MS fragmentation patterns of lobophorin A detected from the liquid culture of *S. olivaceus* JB1 and **(F)** methanol extract from the dried leaf material of *M. japonica*. The arrows indicate exact elution times of the extracted ions.

The methanol extract of the dried foliar residues of *M. japonica* was analyzed with LC/MS chemical profiling. The results clearly showed that lobophorin A (*m*/*z* 1157.6372 [M + H]^+^) was detected at the identical retention time observed in the *S*. *olivaceus* JB1 culture broth extract (Figures 5A–D). Additionally, the MS fragment patterns of the metabolite (*m*/*z* 590.3 [M + H]^+^, 753.4 [M + H]^+^, and 883.5 [M + H]^+^) from two different extracts support the existence of lobophorin A in leaves of *M. japonica* (Figures 5E,F). However, the mass signal intensities were too low to detect the other lobophorin analogs except lobophorin A and G unreported analogs *m*/*z* 997.6 [M + H]^+^ (**18**), and 1141.7 [M + H]^+^ (**20**). In practice, the bacterial cultivation experiment demonstrated a predominant production of lobophorin A from *S*. *olivaceus* JB1 compared to the lobophorin analogs. We also assume that the actual production and accumulation of lobophorin analogs from the endophytic *S*. *olivaceus* JB1 in *M*. *japonica* is limited *in planta*.

### Paper Disk Diffusion Assay

**FIGURE 6:**
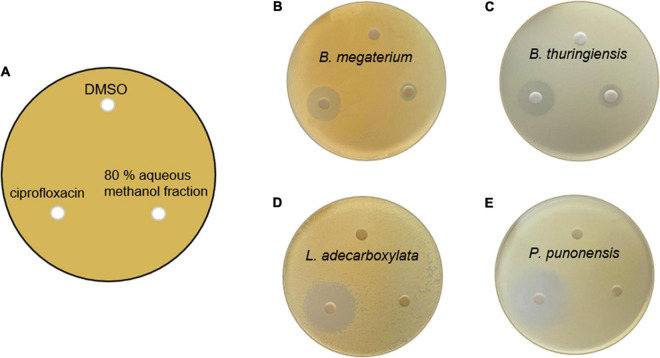
**(A)** Petri plate antibacterial activity assay setup, showing that 500 μL of the four external bacterial contaminants from the leaves of *M*. *japonica* was inoculated and spread on a K agar plate. The placement of lobophorin A and G-containing 80% methanol fraction of *S. olivaceus* JB1 extract dissolved in DMSO (1 mg/mL, 20 μL), the negative control (DMSO only, 20 μL), and the positive control ciprofloxacin dissolved in DMSO (1 mg/mL, 20 μL) 3 days after inoculation. **(B)** Antimicrobial activity against *Bacillus megaterium* JB8; the diameter of growth inhibition of ciprofloxacin was 1.8 cm, and the fraction was 0.9 cm. **(C)** *Bacillus thuringiensis* JB9; the diameter of growth inhibition of ciprofloxacin was 1.9 cm, and the fraction was 1.5 cm. **(D)** *Leclercia adecarboxylata* JB10; the diameter of growth inhibition of ciprofloxacin was 2.5 cm, and the fraction did not show antibacterial activity against *L*. *adecarboxylata.* **(E)** *Pseudomonas punonensis* JB11; the diameter of growth inhibition of ciprofloxacin was 3.0 cm, and the fraction did not show antibacterial activity against *P*. *punonensis*.

The antibacterial activities of lobophorin analogs were previously reported (Niu et al., 2011; Wei et al., 2011; Pan et al., 2013), whereas the analogs did not show antifungal activity. In this study, in the LC/MS system, lobophorin A and G (**2** and **6**) were detected from the botanical sample of *M*. *japonica*, and the compounds were reported to show antibacterial activities in previous studies. We conducted antimicrobial tests with the 80% aqueous methanol fraction containing **2** and **6** from the *S*. *olivaceus* JB1 liquid culture against the four external strains of known phytopathogen strains of *M*. *japonica* (Figure 6). The antibacterial activities of each methanol fraction of the liquid culture were evaluated with diametrical measurement of the inhibition zone. The assay demonstrated that the 80% fraction showed moderate antibacterial activities by suppressing the proliferation of two bacterial contaminants (*Bacillus megaterium* JB8 and *Bacillus thuringiensis* JB9). The diameters of the growth inhibition zones of JB8 and JB9 affected by the 80% methanol fraction were 0.9 and 1.5 cm, respectively (1.8 and 1.9 cm for ciprofloxacin, a positive control), while the fraction did not show antibacterial activity against JB10 (*Leclercia adecarboxylata*) and JB11 (*Pseudomonas punonensis*). An antifungal activity test was performed on agar media containing the fungal inoculum of JB12 (*Mucor circinelloides*), the fungus isolated from the external surface of *M*. *japonica*, with an 80% fraction; however, no antifungal activity was observed. The results demonstrated that the 80% fraction containing lobophorin A and G only showed antibacterial activities against gram-positive *Bacillus* strains.

## Discussion

Endophytes utilize the plant’s endosphere as a unique niche to protect themselves from drastic changes in the external environment (Schindler et al., 2021). Recently, medicinal plants have been reported to be influenced by their interaction with beneficial endophytes (Lindow and Brandl, 2003; Chaudhry et al., 2021). Leaf nodules harboring microbial endophytes in special glandular structures have only recently gained increased interest as examples of plant-microbe interactions in the phyllosphere (Schindler et al., 2021). Detection of the secondary metabolites of symbiotic microorganisms or endophytes have often been reported. For example, the antifungal *Streptomyces* spp. associated with *Protea* infructescences produce fungichromin and actiphenol, and the compounds were detected *in planta* with LC/MS (Human et al., 2016). Antitumor astins were found, with molecular techniques, to originate from the fungal endophyte *Cyanodermella asteris* living within the medicinal plant *Aster tataricus* (Schafhauser et al., 2019).

In this study, we demonstrate the first discovery of seven endophytes of *Maesa japonica* (Thunb.) Moritzi & Zoll. and the presence of lobophorin analogs in the leaf and leaf residues produced by the endophytic bacterium *Streptomyces olivaceus* JB1. At the outset, we postulated that certain isolates may play a pivotal role in defending their host, *M*. *japonica*, against foreign phytopathogens *in planta*, with minimal risk of the plant succumbing to disease. We then isolated seven putative endophytes from *M*. *japonica* – one gram-positive bacterium (*S. olivaceus* JB1), four gram-negative bacteria (*E. ludwigii* JB2, *P. koreensis* JB3, *P. aeruginosa* JB3, and *E. cloacae* JB5) and two fungi (*C. musae* JB6 and JB7) – and five external strains of known phytopathogen strains, including *B. megaterium*, *B. thuringiensis*, *L. adecarboxylata*, *P. punonensis*, and *M. circinelloides*, from the phylloplane to verify the defensive biological system.

Large-scale cultivation of *S*. *olivaceus* JB1 and diverse analyses with LC/MS and NMR data led us to identify the predominant secondary metabolites in the fraction as lobophorin analogs. To track the possible accumulated secondary metabolites produced by the endophytes in *M*. *japonica*, the foliar residues were extracted with methanol, and the extract was analyzed with LC/MS. Interestingly, the lobophorin analogs, mainly lobophorin A and G (**2** and **6**), were detected in the plant material extracts from which the bacterial strain *S*. *olivaceus* JB1 was originally isolated. The detection was confirmed and supported by performing *M*. *japonica* extraction in duplicate, demonstrating the existence of the bacterial strain *S*. *olivaceus* JB1 residing in a leaf microhabitat of *M*. *japonica* as its endophyte and verifying lobophorin analogs accumulation *in situ*.

Among the fractions of the isolate cultures, the 80% aqueous methanol fraction of the bacterial strain JB1 culture displayed moderate antimicrobial activities against two gram-positive bacteria, JB8 and JB9 (*B. megaterium* and *B. thuringiensis*). Lobophorin analogs exhibited strong to moderate antibacterial activities against *M. luteus, M*. *tuberculosis* and *Bacillus* sp. and no activity against *Pseudomonas* sp. which matched antibacterial activity test results (Niu et al., 2011; Luo et al., 2021).

Lobophorins A and B were initially isolated from *Actinomyces* sp. CNB-837 associated with the Caribbean brown alga *Lobophora variegata* (Jiang et al., 1999). Lobophorin analogs belonging to a group of spirotetronate are marine natural products with diverse biological activities. Lobophorin analogs such as L (**1**), A (**2**), M (**3**), H (**4**), E (**5**), G (**6**), K (**7**), CR1 (**8**), C (**9**), I (**10**), and F (**13**) showed antimicrobial activities. For example, lobophorins A, B, E, and F displayed antimicrobial activity against *Bacillus thuringiensis* SCSIO BT01 (Niu et al., 2011), Lobophorin H showed antibacterial activity against *Bacillus subtilis* (Pan et al., 2013). Lobophorin F showed antibacterial activity against St*aphylococcus aureus* ATCC 29213, *Enterococcus faecalis* ATCC 29212 and *Bacillus thuringiensis* SCSIO BT01. Lobophorin K promoted bacteriostatic effects against *Staphylococcus aureus* EPI1167 MSSA. Lobophorins B and F exhibited cytotoxic activity against MCF-7 cells (human breast adenocarcinoma cell line), NCI-H460 (human non-small cell lung cancer cell line), and SF-268 (human glioma cell line) (Niu et al., 2011). Also, lobophorin C represented potent cytotoxic activity against human liver cancer cells, and lobophorin D displayed a significant inhibitory effect on human breast cancer cells (Wei et al., 2011). The case of lobophorin K, the compound represented cytotoxic activity against tumor cell lines such as MIA Paca-2 (pancreatic carcinoma) and MCF-7 (breast adenocarcinoma) (Braña et al., 2017). Lobophorin CR1, CR2, and CR3 showed human oral cancer cell growth inhibition (Cruz et al., 2015). Additionally, potent anti-inflammatory activities of lobophorins A and B were reported (Jiang et al., 1999).

It is currently widely known that lobophorin analogs, spirotetronate polyketides, are produced by marine-associated *Streptomyces* spp. Recently, Rodríguez-Hernández et al. (2019) isolated lobophorin-producing *Streptomyces* sp. from nurse and foraging bees. Here we demonstrate that *Streptomyces olivaceus* JB1 isolated from a terrestrial environment associated with *M*. *japonica* produces lobophorin analogs *in planta*. Environmental transitions of bacteria across the freshwater–marine boundary with a dramatic shift in salinity rarely occur during bacterial evolution (Gophna, 2013). Even though the evolutionary history of the bacterial strain JB1 is currently unknown, we assume that the role of ecological interactions between *M*. *japonica* and *S*. *olivaceus* JB1 in the emergence of the marine and territorial boundary likely diversifies among taxa because of bacterial lifestyle strategies. The results of our salinity manipulation experiment with *S*. *olivaceus* JB1 suggested that the bacterial strain JB1 grows faster and relatively produces more lobophorin analogs in non-saline water. Our LC/MS study indicates that the *S*. *olivaceus* JB1 produces not only known lobophorin analogs produced by marine bacteria but also unreported lobophorin analogs (**14**–**25**) (Supplementary Figure 13). Further lobophorin analog studies are needed with mass bacterial cultivation and chemical diversity analysis based on the backbone of the lobophorin spirotetronate. Furthermore, the biological activity of 12 unreported lobophorin analogs detected only by LC/MS and the ecological functions of the compounds associated with *M*. *japonica* will be examined in the future.

## Data Availability Statement

The datasets presented in this study can be found in online repositories. The names of the repository/repositories and accession number(s) can be found below: NCBI – OM845781, OM845779, OM845778, OM845780, OM838295, OM845784, OM845783, OM838304, OM838312, OM838305, OM838294, and OM845785.

## Author Contributions

SU and SK conceptualized the study. SU collected the plant material, designed the chemical and biological analyses, and identified lobophorin analogs by NMR spectroscopic data. JL did bacterial isolations and fermentations, purified compounds, and performed the biological activity tests. SU, JL, and SK wrote and edited the manuscript. All authors contributed to the article and approved the submitted version.

## Conflict of Interest

The authors declare that the research was conducted in the absence of any commercial or financial relationships that could be construed as a potential conflict of interest.

## Publisher’s Note

All claims expressed in this article are solely those of the authors and do not necessarily represent those of their affiliated organizations, or those of the publisher, the editors and the reviewers. Any product that may be evaluated in this article, or claim that may be made by its manufacturer, is not guaranteed or endorsed by the publisher.

## Footnotes

1
http://gnps.ucsd.edu

## Abbreviations

LC/Q-TOF-MS: liquid chromatography-quadrupole-time of flight mass spectrometer
HPLC: high-performance liquid chromatography
GNPS: Global Natural Product Social Molecular Networking
TIC: total ion chromatogram
EIC: extracted ion current chromatogram
NMR: nuclear magnetic resonance
COSY: correlation spectroscopy
HSQC: heteronuclear single quantum coherence
HMBC: heteronuclear multiple bond correlation.
