# Supplementary material for: Lobophorin Producing Endophytic Streptomyces olivaceus JB1 Associated With Maesa japonica (Thunb.) Moritzi & Zoll
Source: Front Microbiol. 2022 Apr 29;13:881253. doi: 10.3389/fmicb.2022.881253 (PMC9100408; doi:10.3389/fmicb.2022.881253)
Supplement: Supplementary file 1 [file Data_Sheet_1.PDF]

Supplementary Material

Lobophorin Producing Endophytic *Streptomyces olivaceus* JB1 Associated with *Maesa japonica* (Thunb.) Moritzi & Zoll

Soohyun Um[1†], Jaeyoun Lee [1†], Seung Hyun Kim[1,*]

[1] College of Pharmacy, Yonsei Institute of Pharmaceutical Sciences, Yonsei University, Incheon 21983, South Korea

Corresponding authors:
*(S.H.K.) Tel: +82-32-749-4514. E-mail: kimsh11@yonsei.ac.kr

Table of Contents

S3 : **Supplementary Figure 1.** A comparison of two different culture conditions.

S4 : **Supplementary Figure 2.** ^{1}H NMR spectrum (700 MHz) of lobophorin A in CDCl3-d1/CD3OD-d4 solvent mixture.

S5 : **Supplementary Figure 3.** COSY NMR spectrum (700 MHz) of lobophorin A in $CDCl_3$-d_1/CD_3OD-d_4 solvent mixture.
Supplementary Figure 4. HSQC NMR spectrum (700 MHz) of lobophorin A in $CDCl_3$-d_1/CD_3OD-d_4 solvent mixture.

S6 : **Supplementary Figure 5.** HMBC NMR spectrum (700 MHz) of lobophorin $CDCl_3$-d_1/CD_3OD-d_4 solvent mixture.

S7 : **Supplementary Figure 6.** ^{1}H NMR spectrum (800 MHz) of lobophorin G in DMSO-d_6.
Supplementary Figure 7. ^{13}C NMR spectrum (200 MHz) of lobophorin G in DMSO-d_6.

S8 : **Supplementary Figure 8.** COSY NMR spectrum (800 MHz) of lobophorin G in DMSO-d_6.
Supplementary Figure 9. HSQC NMR spectrum (800 MHz) of lobophorin G in DMSO-d_6.

S9 : **Supplementary Figure 10.** HMBC NMR spectrum (800 MHz) of lobophorin G in DMSO-d_6.
Supplementary Figure 11. TOCSY NMR spectrum (800 MHz) of lobophorin G in DMSO-d_6.

S10 : **Supplementary Figure 12.** The pictures of *Maesa japonica* (Thunb.) Moritzi & Zoll.

S11 : **Supplementary Figure 13.** Total ion current (TIC) chromatogram and Extracted-ion chromatogram (EIC) of the lobophorin analogues detected from the liquid broth of *S. olivaceus* JB1.

1.1 Supplementary Figures

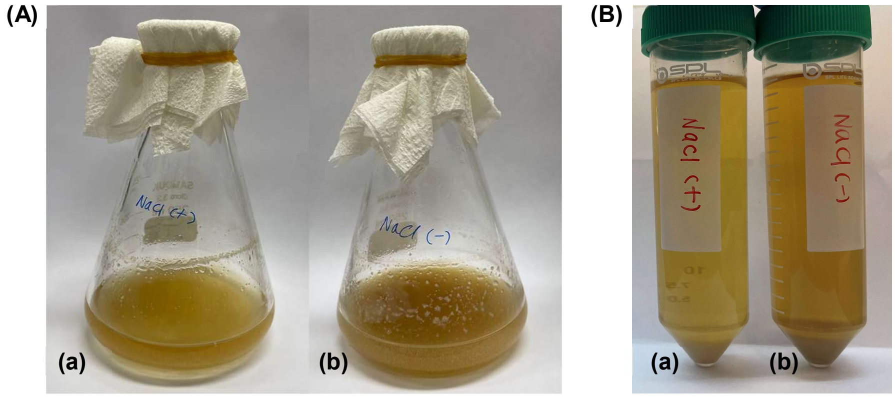

Supplementary Figure 1. A comparison of two different culture conditions. (A) Culture broth of *S. olivaceus* JB1 for 4 days. (B) After centrifugation with 4000 rpm for 10 minutes of the liquid culture of *S. olivaceus* JB1 for 4 days. (a) Culture for 4 days of *S. olivaceus* JB1 in saline media (salinity; 33 per mille of sodium chloride) (b) Culture for 4 days of *S. olivaceus* JB1 in non-saline. After centrifugation, precipitated cells were weighed. (a) 1.61 g and (b) 2.67 g.

Supplementary Figure 2. ^{1}H NMR spectrum (700 MHz) of lobophorin A in $CDCl_3$-d_1/ CD_3OD-d_4 solvent mixture.

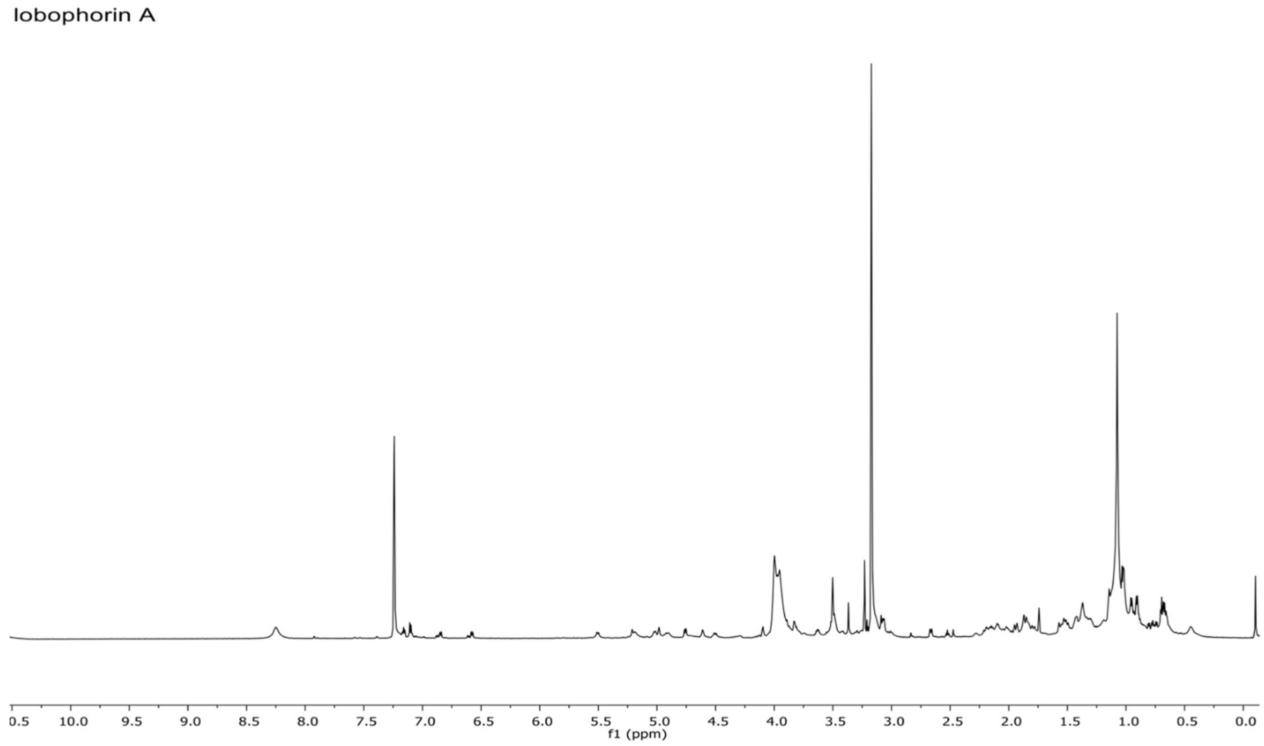

Supplementary Figure 3. COSY NMR spectrum (700 MHz) of lobophorin A in $CDCl_3$-*d*1/CD_3OD-*d*4 solvent mixture.

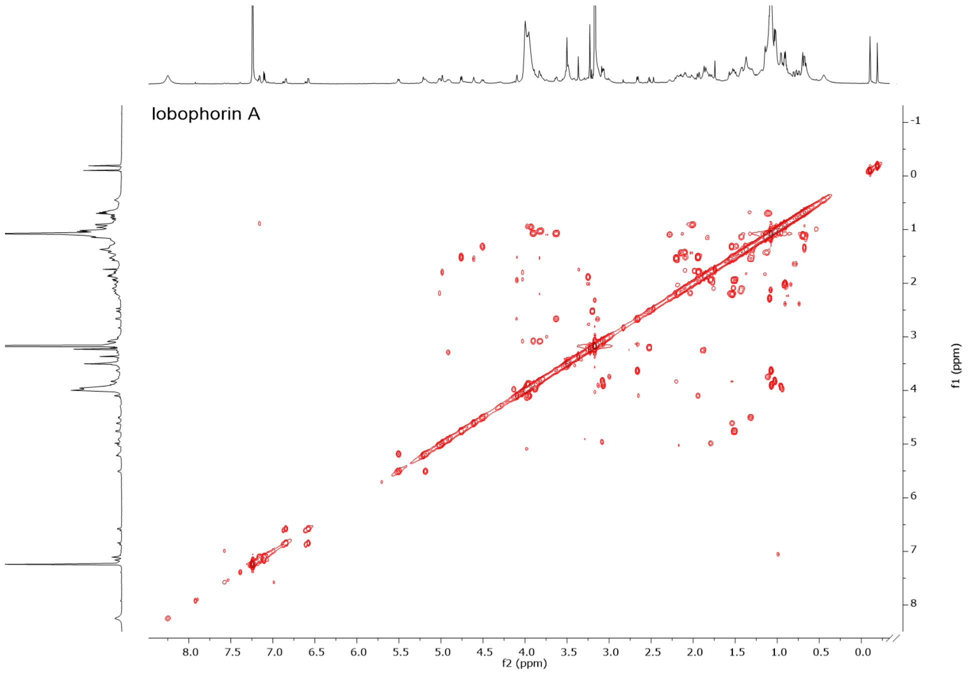

Supplementary Figure 4. HSQC NMR spectrum (700 MHz) of lobophorin A in $CDCl_3$-*d*1/ CD_3OD-*d*4 solvent mixture.

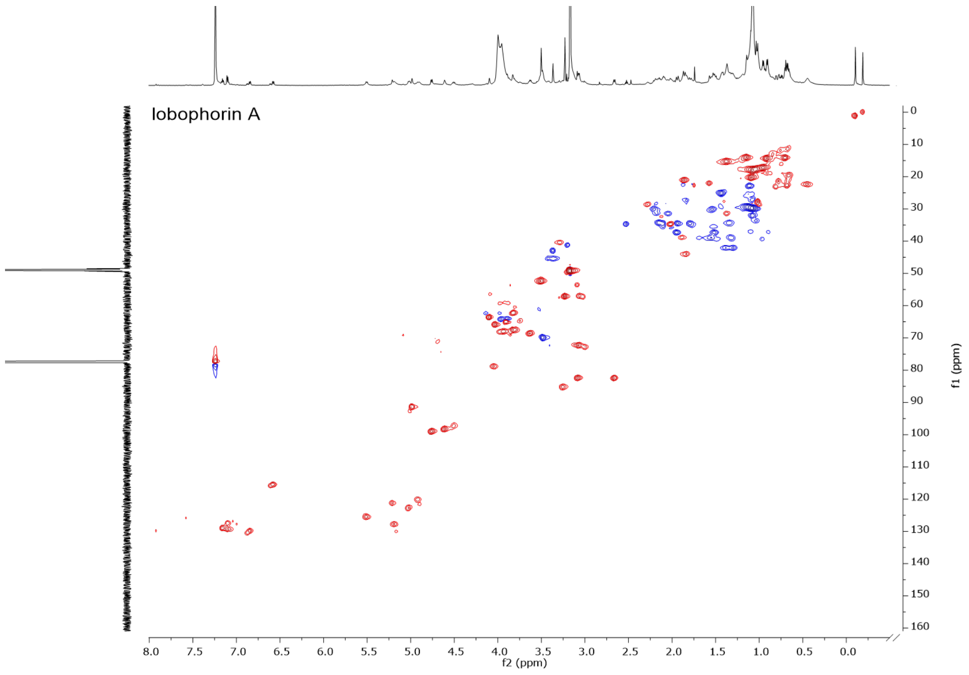

Supplementary Figure 5. HMBC NMR spectrum (700 MHz) of lobophorin A in $CDCl_3$-d_1/ CD_3OD-d_4 solvent mixture.

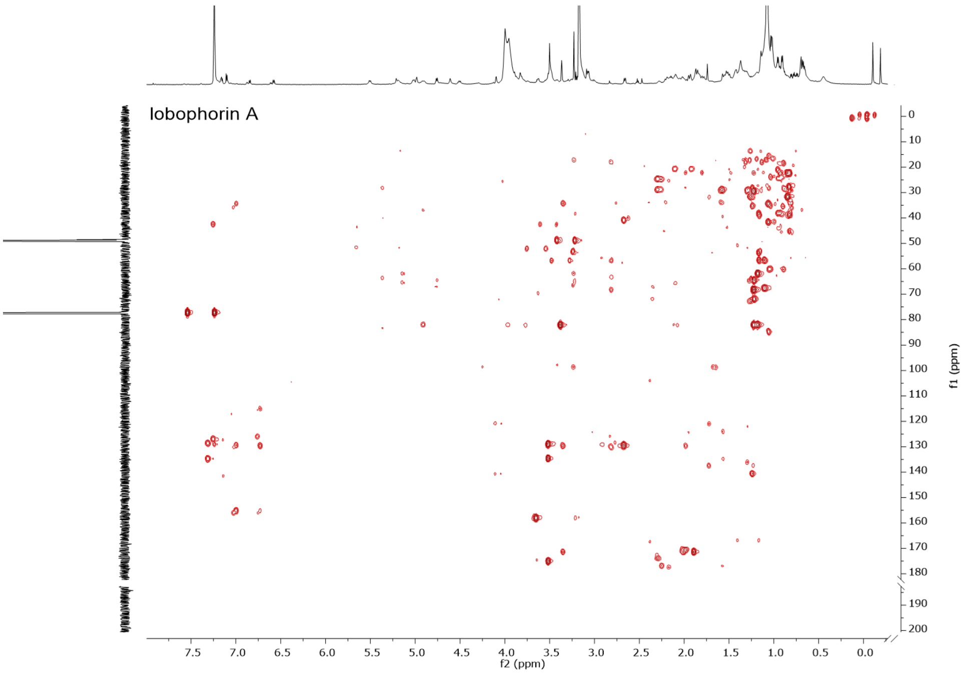

Supplementary Figure 6. ^{1}H NMR spectrum (800 MHz) of lobophorin G in DMSO-d_6.

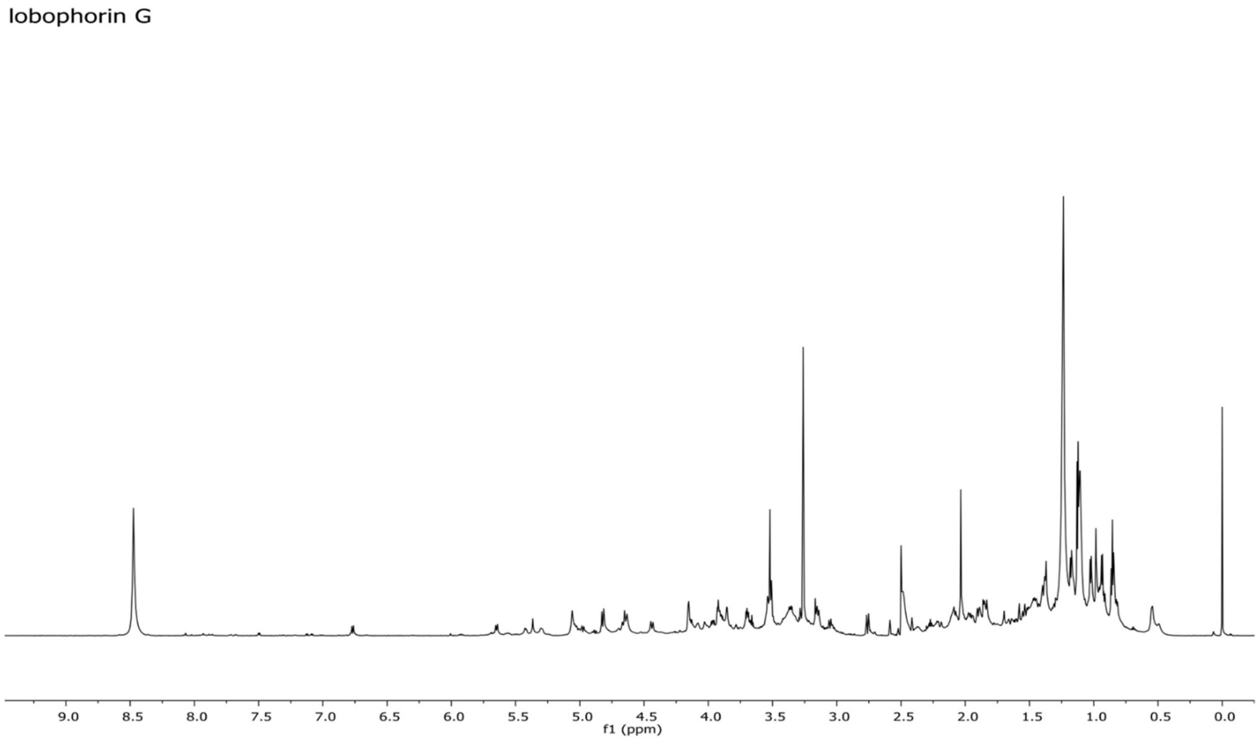

Supplementary Figure 7. ^{13}C NMR spectrum (200 MHz) of lobophorin G in DMSO-d_6.

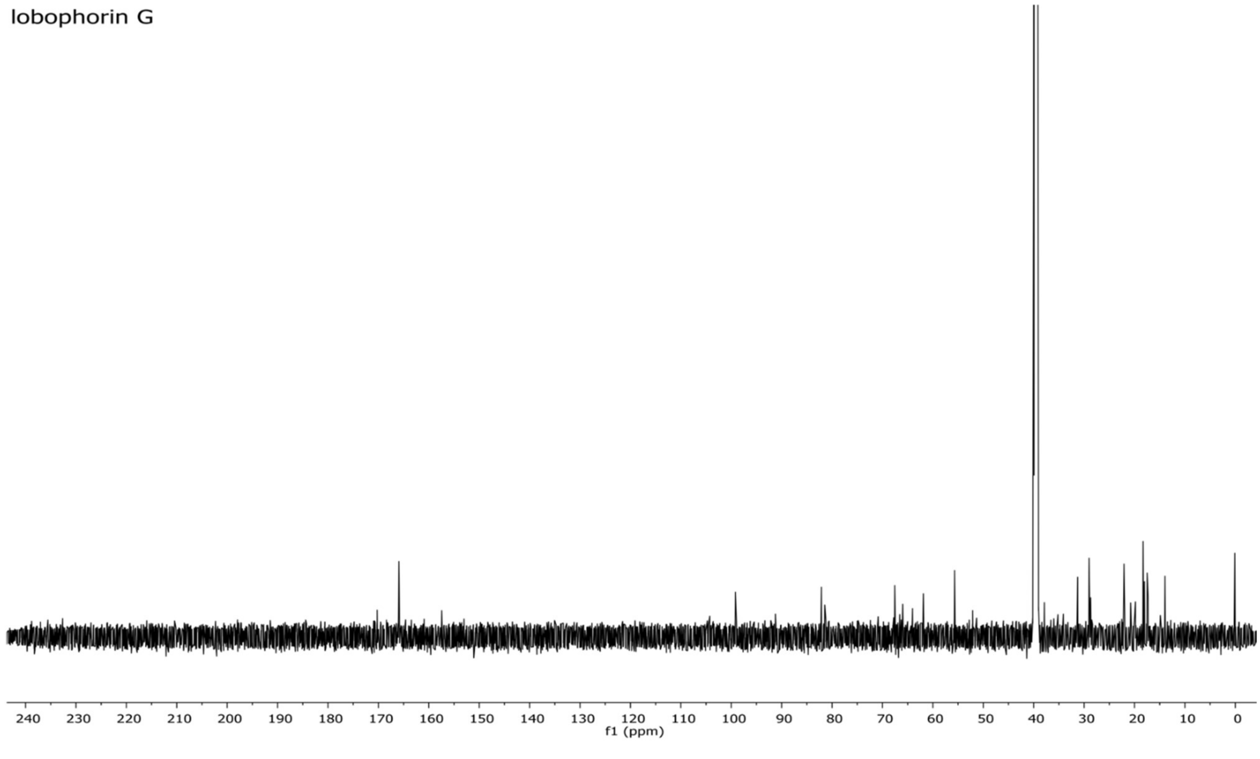

Supplementary Figure 8. COSY NMR spectrum (800 MHz) of lobophorin G in DMSO-d_6.

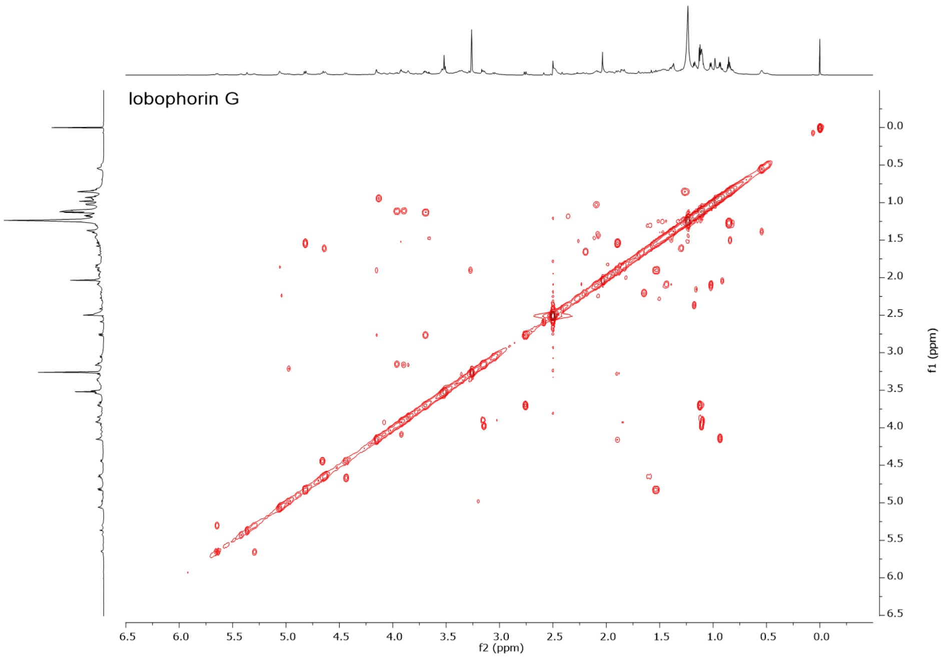

Supplementary Figure 9. HSQC NMR spectrum (800 MHz) of lobophorin G in DMSO-d_6.

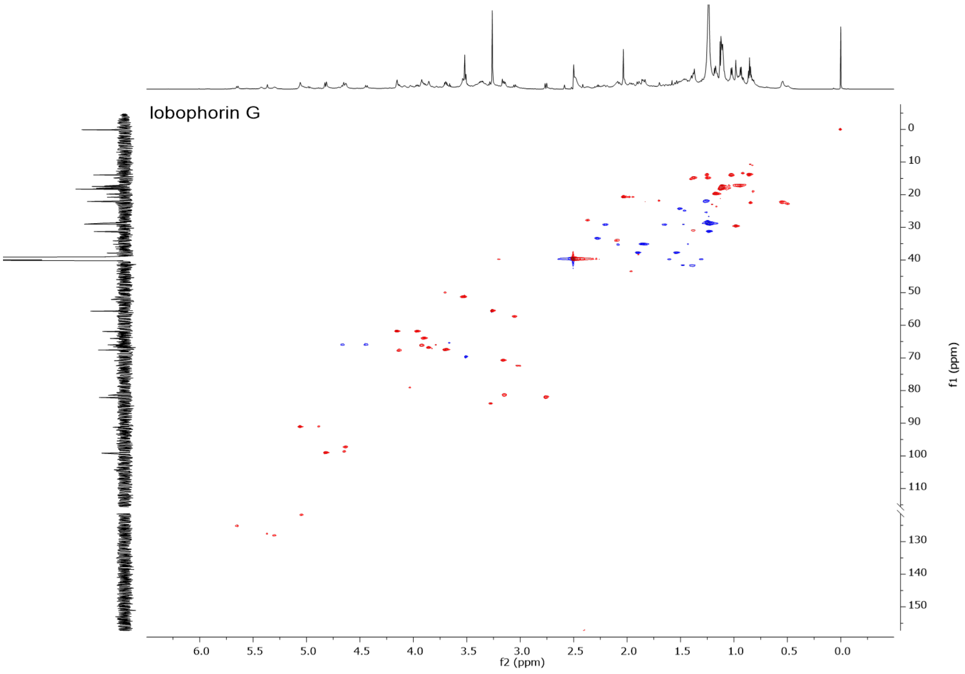

Supplementary Figure 10. HMBC NMR spectrum (800 MHz) of lobophorin G in DMSO-d_6.

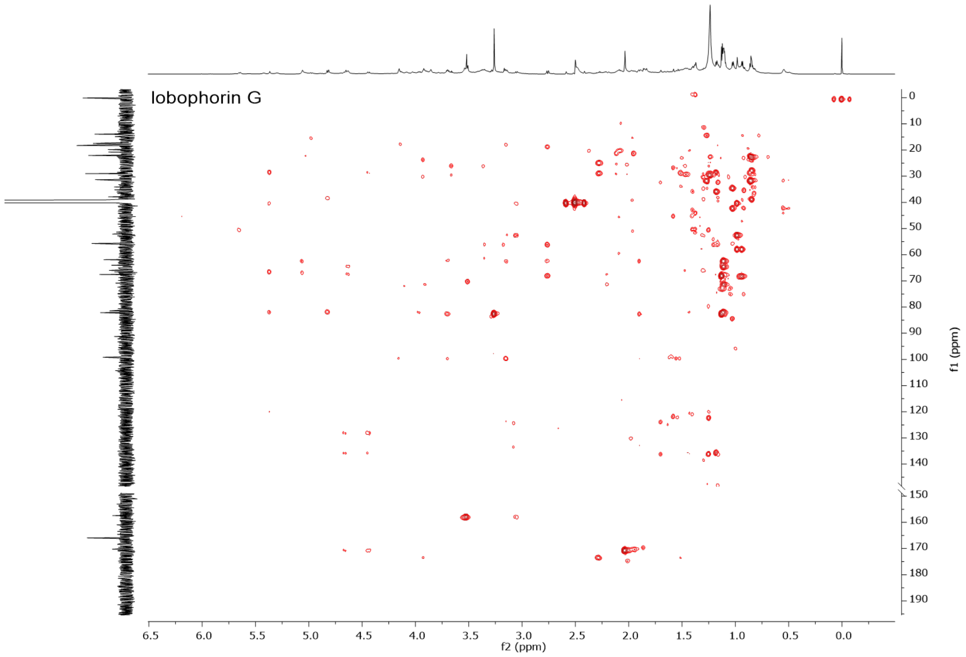

Supplementary Figure 11. TOCSY NMR spectrum (800 MHz) of lobophorin G in DMSO-d_6.

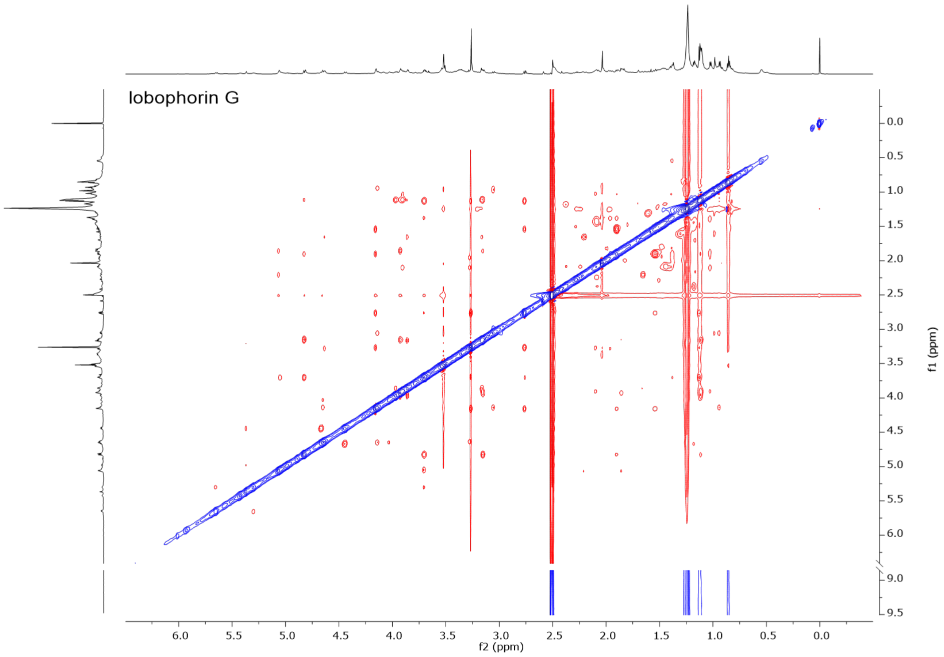

Supplementary Figure 12. The pictures of *Maesa japonica* (Thunb.) Moritzi & Zoll. **(A)** Aerial parts of *M. japonica* from Jeju Island, Korea. **(B)** The leaves, leaf residues and branches of *M. japonica.*

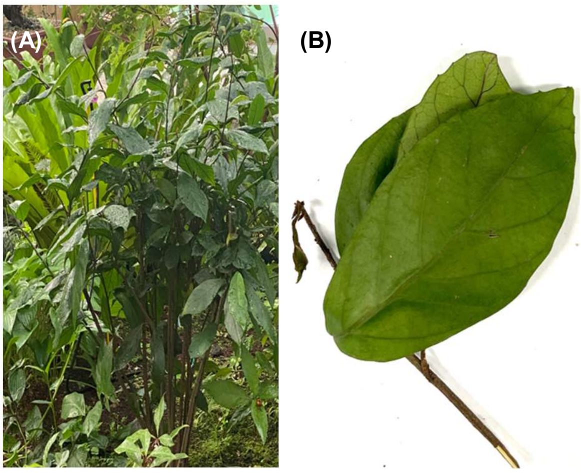

Supplementary Figure 13. (A) Total ion current (TIC) chromatogram of liquid culture broth of *S. olivaceus* JB1. (B) Extracted-ion chromatogram (EIC) of the known lobophorin analogues detected from the liquid broth of *S. olivaceus* JB1. (C) Extracted-ion chromatogram (EIC) of the unknown lobophorin analogues detected from the liquid broth of *S. olivaceus* JB1.

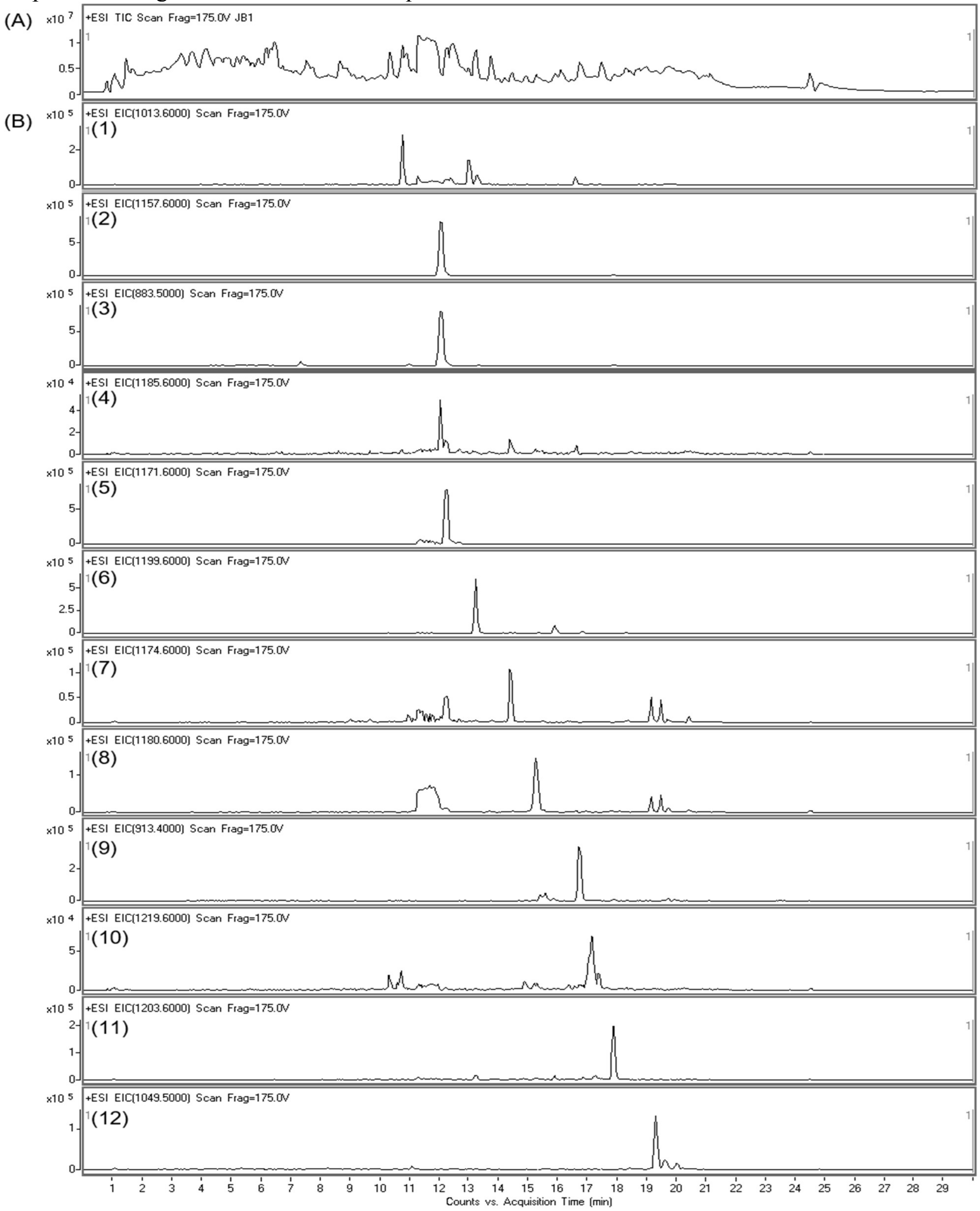

(C)

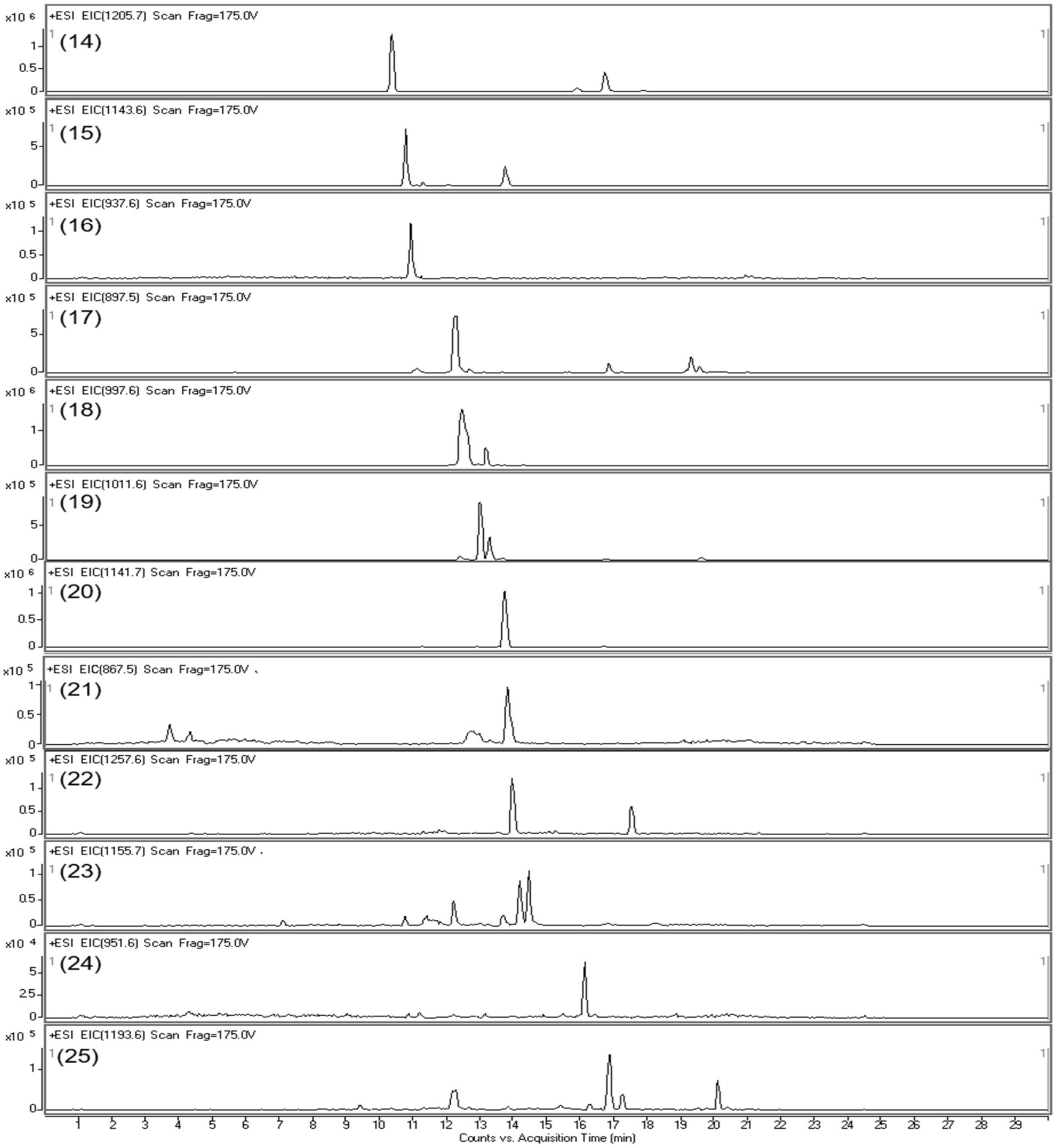

+ESI EIC(1205.7) Scan Frag=175.0V
(14)
+ESI EIC(1143.6) Scan Frag=175.0V
(15)
+ESI EIC(937.6) Scan Frag=175.0V
(16)
+ESI EIC(897.5) Scan Frag=175.0V
(17)
+ESI EIC(997.6) Scan Frag=175.0V
(18)
+ESI EIC(1011.6) Scan Frag=175.0V
(19)
+ESI EIC(1141.7) Scan Frag=175.0V
(20)
+ESI EIC(867.5) Scan Frag=175.0V
(21)
+ESI EIC(1257.6) Scan Frag=175.0V
(22)
+ESI EIC(1155.7) Scan Frag=175.0V
(23)
+ESI EIC(951.6) Scan Frag=175.0V
(24)
+ESI EIC(1193.6) Scan Frag=175.0V
(25)
Counts vs. Acquisition Time (min)
